# Draft genome *of Gemmata massiliana* sp. nov, a water-borne *Planctomycetes* species exhibiting two variants

**DOI:** 10.1186/s40793-015-0103-0

**Published:** 2015-12-07

**Authors:** Rita Aghnatios, Caroline Cayrou, Marc Garibal, Catherine Robert, Said Azza, Didier Raoult, Michel Drancourt

**Affiliations:** Aix-Marseille Université, URMITE, UM63, CNRS7278, IRD198, Inserm 1095, Faculté de médecine, 27 Boulevard jean Moulin, 13385 Marseille, cedex 05, France

**Keywords:** *Gemmata massiliana*, *Gemmata obscuriglobus*, *Gemmata*, *Planctomycetes*, Hospital water network, Culture, Genome

## Abstract

*Gemmata massiliana* is a new *Planctomycetes* bacterium isolated from a hospital water network in France, using a new culture medium. It is an aerobic microorganism with optimal growth at pH 8, at 30 °C and salinity ≤ 1.25 % NaCl. *G. massiliana* is resistant to β-lactam antibiotics, due to lack of peptidoglycan in its cell wall.*G. massiliana* shares a 97 % 16S rRNA gene sequence similarity with the nearest species, *Gemmata obscuriglobus;* and 99 % similarity with unnamed soil isolates*.* Its 9,249,437-bp genome consists in one chromosome and no detectable plasmid and has a 64.07 % G + C content, 32.94 % of genes encoding for hypothetical proteins. The genome contains an incomplete 19.6-kb phage sequence, 26 CRISPRs, 3 CAS and 15 clusters of secondary metabolites. *G. massiliana* genome increases knowledge of a poorly known world of bacteria.

**Electronic supplementary material:**

The online version of this article (doi:10.1186/s40793-015-0103-0) contains supplementary material, which is available to authorized users.

## Introduction

*Gemmata obscuriglobus**,* the sole cultured representative of the genus *Planctomycetes**Gemmata*, was first isolated in a freshwater dam in Queensland, Australia [1]. *Gemmata* isolates were thereafter cultivated from the leakage water of a compost heap [2], an Australian soil specimen and freshwater [3] and sphagnum peat bogs [4]. Moreover, 16S rRNA gene sequences of *Gemmata* were detected in various environments, including a municipal wastewater treatment plant [5], rivers in Germany [6], soil [7], sphagnum peat bogs [4], clean rooms where spacecraft are assembled [8], a water specimen of the Western Pacific Ocean and sediments [9], a South African water spring [10], the gastrointestinal tract of carp [11], nonsulfur, sulfur and iron geothermal steam vents [12] and recently from human stool specimens [13].

*G. obscuriglobus* exhibits intriguing features, including a condensed chromatin that is surrounded by a double membrane, a rare feature among bacteria, which evokes a nucleus-like compartmentalization [14]. It has been recently debated that membrane invaginations may be the actual cause of this intracellular membranous organization [15]. Moreover, *G. obscuriglobus* is remarkable for its capacity to survive high doses of ionizing radiation and ultraviolet light at energy values generally depicting an ability to maintain genomic integrity [16]. It also possesses membrane coat-like proteins that are implicated in endocytosis-like processes, a feature long thought to be exclusive to eukaryotes [17]. *G. obscuriglobus* exhibits a large number of extracytoplasmic function sigma factors illustrating its skilled adaptation to stress and reactivity to environmental stimulus [18]. *G. obscuriglobus* shared many eukaryotic homologous genes including a homolog of integrin alpha-V which is implicated in signal transduction and cytoskeleton organization [19].

Herein, we describe a new isolate as a representative of a new species *Gemmata massiliana*, with the aim of enlarging the scope of our knowledge regarding this fascinating bacterial genus. This isolate, which has 99 % 16S rRNA gene similarity with Australian soil and freshwater strains which have been isolated but neither described nor sequenced [3], was this time isolated from a hospital water distribution system. Evidently, the choice of the culture medium had a primary effect on the growth of this bacterium [20]. It was actually elaborated using some of the filtered sample water itself as a medium basis that could simulate the natural environment and provide the microorganism with the necessary chemical components for its growth. Furthermore, antibiotics were added to the culture medium for a selective isolation of *Planctomycetes* which are broadly resistant to antibiotics [21]. Phenotypic and genomic features of *G. massiliana* sp. nov. strain CSUR P189^T^ are presented hereafter.

## Organism information

### Classification and features

**Fig. 1:**
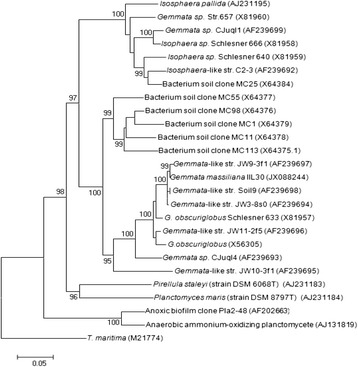
*Gemmata massiliana* phylogenetic position amongst other *Planctomycetes* species. The following Phylogenetic tree shows *G. massiliana* strain CSUR P189^T^ position relatively to *Gemmata obscuriglobus*, to undescribed *Gemmata* species and to other *Planctomycetes*. Sequences were aligned using CLUSTALW [39], and phylogenetic inferences obtained using the neighbor-joining method within the MEGA software [40] only bootstrap values ≥95 % are indicated at nodes. *T. maritima* (M21774) was used as an outgroup. The scale bar represents a 5 % nucleotide sequence divergence

From September 2011 to August 2012, 15 points located along the water network in two hospitals in Marseille, France were sampled on a weekly basis. Water samples were collected into sterile, 500-mL containers (Dominique Dutscher, Brumath, France) containing sodium thiosulfate used to neutralize free chlorine. The water specimens were inoculated on the same day of the sampling into the Marine-like (ML) and *Isosphaera*-like (IL) enrichment broths incubated at 22 °C and 30 °C, in the presence of negative controls (enrichment broth without water sample) as previously described [22]. The enrichment broth consisted of the specimen water itself, passed through a 0.2-μm membrane filter (Thermo Fisher Scientific, Saint Herblain, France) complemented with a 10 % vol/vol antibiotic solution containing 40 mg/L vancomycin, 100 mg/L imipenem, 1 mg/L penicillin G and 32 mg/L amphotericin B; in addition to an enrichment solution (5 g of peptone and 1 g of yeast extract per 100 mL) for ML broth and a vitamin solution for the IL broth made of 60 μg β-aminobenzoic acid, 6 μg biotin, 3 μg vitamin B12, 600 μg nicotinamide, 300 μg thiamin, 150 mg glucose and 150 mg peptone from casein per liter of specimen filtered water (Sigma-Aldrich, Saint-Quentin Fallavier, France). A 2 mL-volume of the water sample was centrifuged at 17,000 × g using the Heraeus Pico 17 centrifuge (Thermo Fisher Scientific) for 5 min and the pellet was inoculated into 5 mL of the enrichment broth. Presence of any turbidity was monitored daily for four months. Once the turbidity was detected, 10 μL of inoculated broth were spread on solid medium that had the same composition as broth, complemented with 1.5 % agar (Sigma-Aldrich). All colonies were identified by matrix-assisted laser-desorption/ionization time-of-flight mass spectrometer (MALDI-TOF-MS) (Bruker Daltonics, Bremen, Germany) as previously described [23]. Further identification was based on 16S rRNA gene PCR amplification and sequencing [13]. Observations by electron microscopy were done as previously described [24]. Briefly, the bacteria were suspended and then washed in phosphate buffer and stained with 1 % (w/v) phosphotungstic acid. Afterwards examination was carried on using Morgagni 268D (Philips) electron microscope at an operating voltage of 60 kV. Also, a 10^6^ bacterial suspension of *G. massiliana* was examined for cell size variation using BD LSRFortessa cell analyzer (Becton Dickinson, Le Pont de Claix, France) and FACSDiva software (version 6.2) as previously described [25]. Further characterization of the isolate comprised the observation of growth under anaerobic, aerobic, microaerophilic and presence of 5 % CO_2_ atmosphere; inoculation of Api 20E, 20NE, ZYM, and 50CH strips, (bioMérieux, La Balme les grottes, France) E-test (bioMérieux), pH, salinity and temperature tolerance. *G. massiliana* strain CSUR P189^T^ sequenced in this study (Table 1) was isolated in December 2011 after 2-month incubation at 30 °C in *Isosphaera*-like agar preceded by 4 weeks incubation in *Isosphaera*-like broth. MALDI-TOF-MS yielded insignificant scores below 0.3. This isolate exhibited 97 % 16SrRNA gene nucleotide sequence (GenBank accession number JX088244) similarity with *G. obscuriglobus* (GenBank accession number X81957), a value lower than the threshold that was defined by Stackebrandt and Ebers to depict a new species [26]. And as stated above, *G. massiliana* also displayed 16S rRNA gene nucleotide sequence similarity of 99 % with unnamed isolates [3]. Those bacteria are most likely various *G. massiliana* strains (Fig. 1).

**Table 1:** Classification and general features of *Gemmata massiliana* strain IIL30^T^

MIGS ID	Property	Term	Evidence code^a^
	Classification	Domain: *Bacteria*	TAS [41]
		Phylum: *Planctomycetes*	TAS [42]
		Class: *Planctomycetia*	TAS [43]
		Order: *Planctomycetales*	TAS [43]
		Family: *Planctomycetaceae*	TAS [44]
		Genus: *Gemmata*	TAS [1]
		Species: *Gemmata massiliana*	IDA
		Type strain: IIL30 CSUR P189^T^	IDA
	Gram stain	Negative	IDA
	Cell shape	Coccus	IDA
	Motility	Motile	IDA
	Sporulation	Nonsporulating	IDA
	Temperature range	Mesophile	IDA
	Optimum temperature	30°C	IDA
	pH range; Optimum	6–8; 8	IDA
	Carbon source	Unknown	
GS-6	Habitat	Hospital water	IDA
MIGS-6.3	Salinity	0 % NaCl (w/v)	IDA
MIGS-22	Oxygen requirement	Aerobic	IDA
MIGS-15	Biotic relationship	Free-living	IDA
MIGS-14	Pathogenicity	Unknown	
MIGS-4	Geographic location	France/Marseille	IDA
MIGS-5	Sample collection	October 2011	IDA
MIGS-4.1	Latitude	43.3	IDA
MIGS-4.2	Longitude	5.4	
MIGS-4.4	Altitude	Unknown	

^a^ Evidence codes - *IDA* Inferred from Direct Assay, *TAS* Traceable Author Statement (i.e., a direct report exists in the literature), *NAS* Non-traceable Author Statement (i.e., not directly observed for the living, isolated sample, but based on a generally accepted property for the species, or anecdotal evidence). These evidence codes are from the Gene Ontology project [45]

**Fig. 2:**
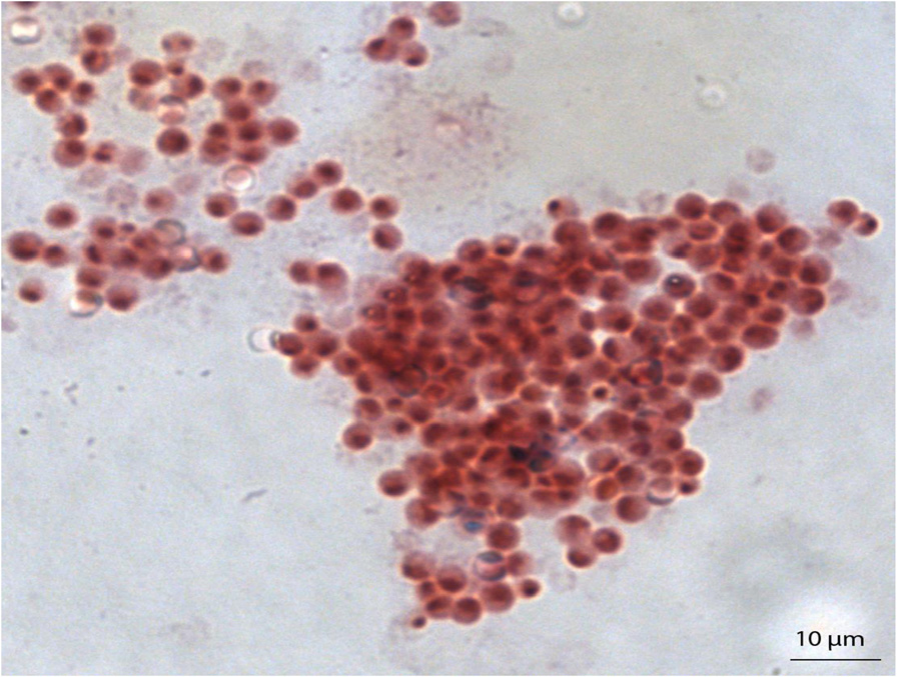
Gram staining of *G. massiliana* strain CSUR P189^T^. The bar scale reprensents 10 μm

**Fig. 3:**
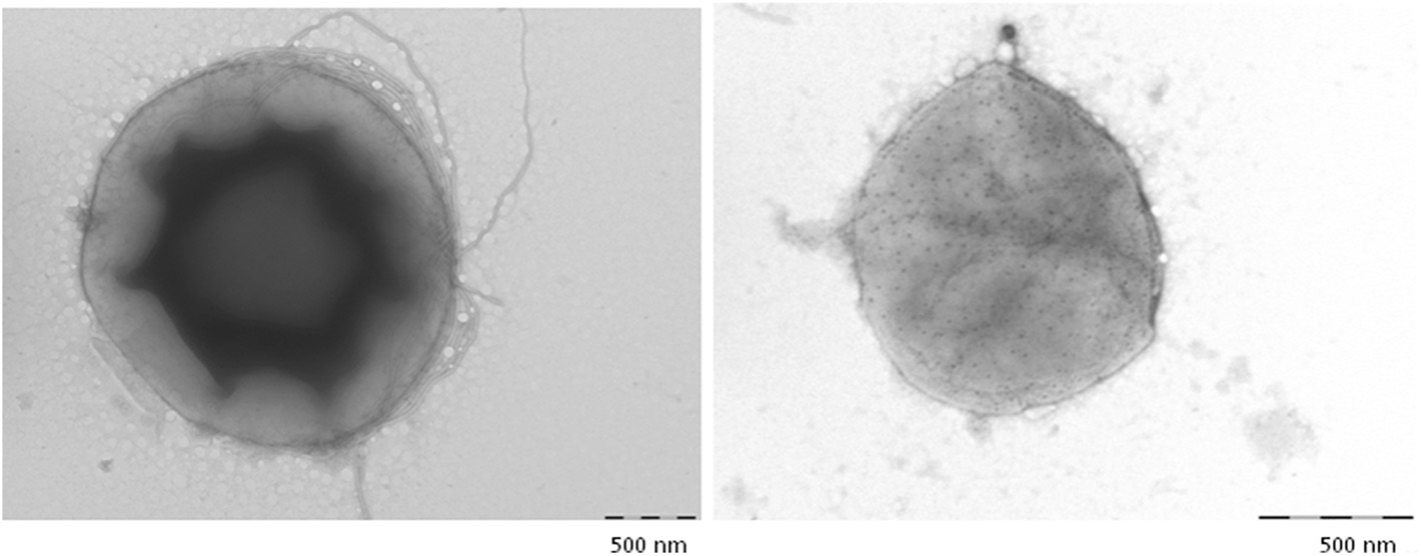
*G. massiliana* transmission electron microscopy. We observed two size-different populations of *G. massiliana* strain CSUR P189^T^, using a Morgagni 268D (Philips) at an operating voltage of 60kV. The scale bar represents 500 nm

**Fig. 4:**
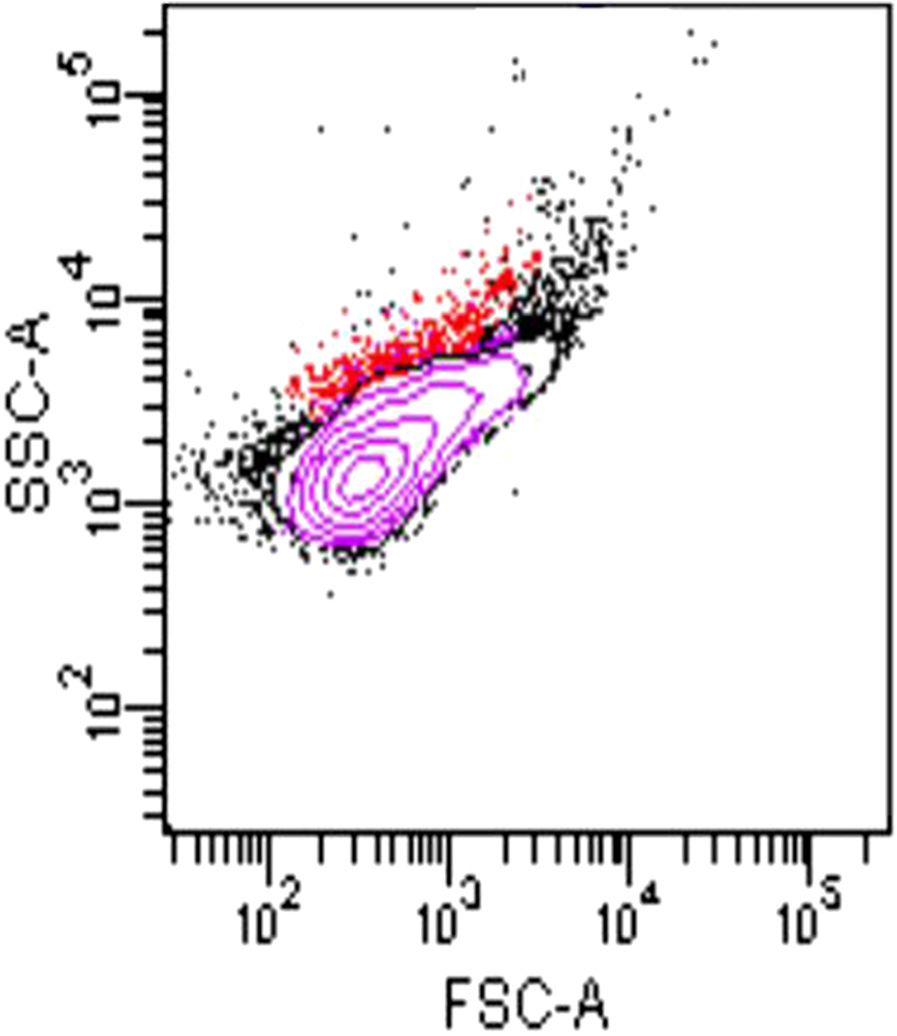
*G. massiliana* two cell variants as demonstrated by flow cytometry. Representative histogram of the two cell variants in a *G. massiliana* sample shown in purple, was generated by a BD LSRFortessa (Le Pont de Claix, France) and FACSDiva software (version 6.2)

**Fig. 5:**
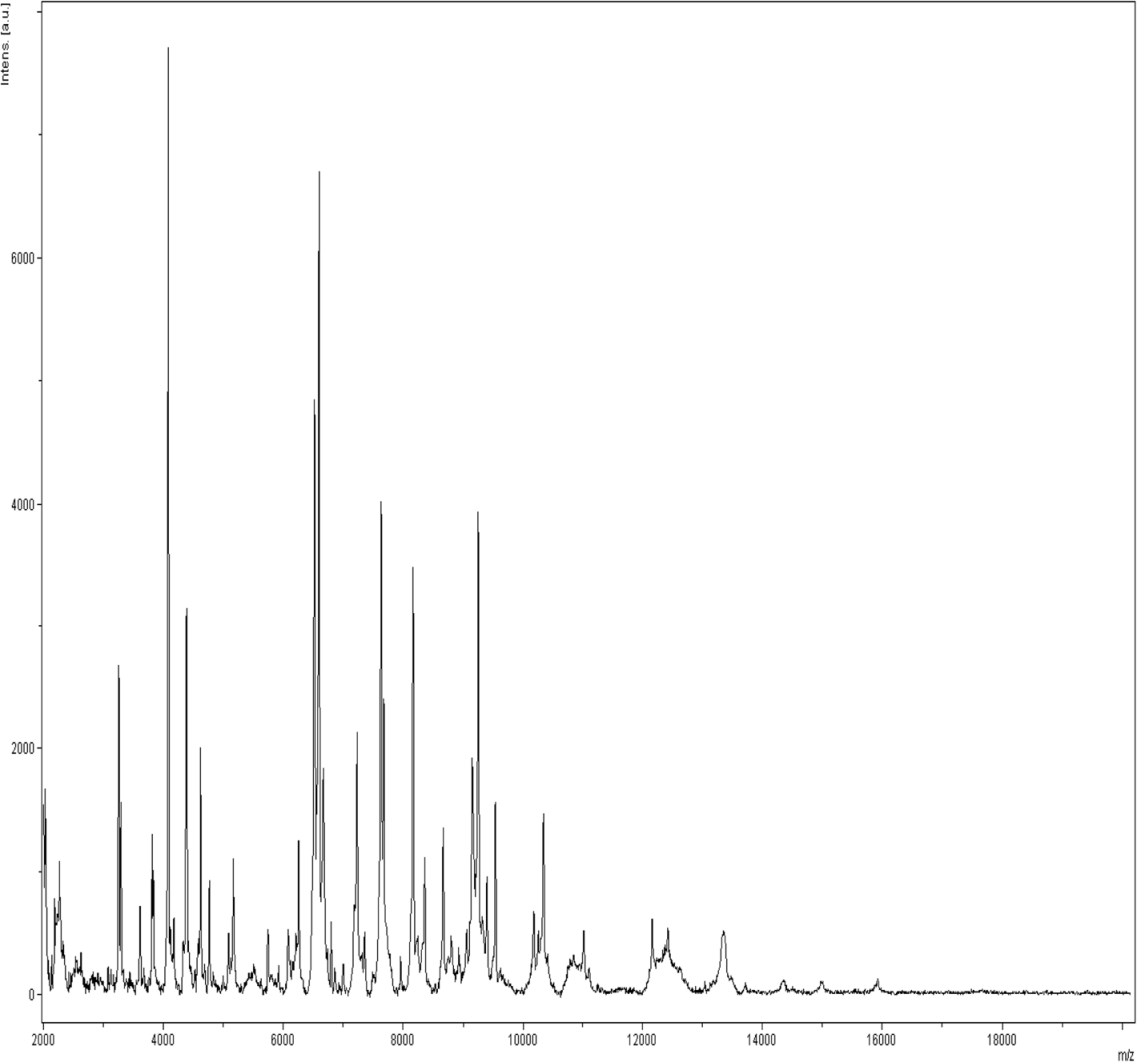
Reference mass spectrum of *G. Massiliana* strain CSUR P189^T^. This reference spectrum was generated from 10 spectra corresponding to 10 deposited colonies of *G. massiliana*

In all culture-based observations, the negative controls remained sterile. *G. massiliana* grew at 25 °C, 30 °C and 37 °C; no growth was observed at 4 °C or at 45 °C, and growth was optimal at 30 °C. The diameter of the colonies varied between 0.1 mm and 1 mm on *Isosphaera*-like agar. Growth was observed in all tested atmospheres for the exception of the anaerobic atmosphere, optimal growth occurred in aerobic conditions. Tolerable salinity varied between 0 and 1.25 % with an optimal growth in the absence of salt; pH tolerance varied from pH 6 to pH 8, with an optimal growth at pH 8. Cells grown on agar are motile and Gram-negative (Fig. 2). Negative staining showed two populations of cells, including a small-cell-variant with a 1.1-μm diameter and a large-cell variant with a 2.1-μm diameter (Fig. 3). This feature is not an electron microscopy artifact since FACS scan further disclosed two populations within the *G. massiliana* cultured on *Caulobacter* agar for 7 days (Fig. 4). The isolate tested negative for catalase and oxydase and positive for esculinase, alkaline phosphatase, naphtol-AS-BI-phosphohydrolase, valine, trypsin, acid phosphatase and leucine arylamidase. It was resistant to β-lactam antibiotics at concentrations of 32 mg/L for penicillin G and imipenem, 256 mg/L for vancomycin and 66 mg/L for amphotericin B. *G. massiliana* exhibited intermediate susceptibility to chloramphenicol (MIC, 6 mg/L), colistin (MIC, 6 mg/L) and is susceptible to tetracycline (MIC, 0.016 mg/L), ciprofloxacin (MIC, 0.016 mg/L) cotrimoxazole (MIC, 0.016 mg/L), gentamicin (MIC, 0.016 mg/L), minocycline (MIC, 0.016 mg/L), erythromycin (MIC, 0.016 mg/L) and rifampicine (MIC, 0.016 mg/L). Matrix-assisted laser-desorption/ionization time-of-flight mass spectrometer (MALDI-TOF-MS) yielded a unique peptidic profile (Fig. 5). The isolate has been deposited in two public collections as *G. massiliana* strain IIL30^T^, in the Collection de Souches de l’Unité des Rickettsies (Marseille, France; CSUR P189^T^) and Deutsche Sammlung von Mikrorganismen and Zellkuturen (Braunshwing, Germany; DSM 26013^T^).

## Genome sequencing information

### Genome project history

The organism was selected for sequencing on the basis of its phylogenetic position and 16S rRNA gene similarity to *G. obscuriglobus**,* the sole named species in this genus, and its isolation was done in the context of a study on the detection of *Planctomycetes* bacteria in the hospital water network. The bioproject Genbank accession number is PRJEB621 and consists of 417 contigs and 22 scaffolds. Table 2 shows the project information and its association with MIGS version 2.0 compliance [27].

**Table 2:** Project information

MIGS ID	Property	Term
MIGS-31	Finishing quality	Draft
MIGS-28	Libraries used	454 paired end 3-kb and shotgun XL+ libraries
MIGS-29	Sequencing platforms	454 Roche Titanium
MIGS-31.2	Fold coverage	22.4×
MIGS-30	Assemblers	Newbler version 2.8
MIGS-32	Gene calling method	CLC genomics workbench 6.0.1
	Genbank ID	CBXA000000000.1
	Genbank Date of Release	January 20,2014
	GOLD ID	Gp0033443
	BIOPROJECT	PRJEB621
MIGS-13	Source Material Identifier	DSM 26013
	Project relevance	Medical, hospital water network

### Growth conditions and genomic DNA preparation

*G. massiliana* strain IIL30 was grown aerobically on *Caulobacter* agar (peptone enzymatic hydrolysate type I: from meat; 2 g/L, yeast extract; 1 g/L, MgSO_4_; 0.2 g/L, agar; 15 g/L) at 30 °C. A 300 μL-bacterial suspension was diluted in 1 mL TE buffer for lysis treatment: a lysozyme incubation of 30 min at 37 °C followed by an overnight Proteinase K incubation at 37 °C. The DNA was purified by three phenol-chloroform extractions and ethanolic precipitation at -20 °C overnight. After centrifugation, the DNA was resuspended in 144 μL TE buffer. The concentration was measured by the Quant-it Picogreen kit (Invitrogen, Saint Aubin, France) on the Genios_Tecan fluorometer at 67.4 ng/μl.

### Genome sequencing and assembly

A shotgun XL+ and 3-kb paired-end libraries were pyrosequenced on the 454_Roche_Titanium. This project was loaded twice on a 1/4 region for the paired end application on PTP Picotiterplate and once ¼ region for the shotgun XL+ strategy. The XL+ shotgun library was constructed with 1μg of DNA as described by the manufacturer (Roche, Meylan, France) with the GS Rapid library XL+ Prep kit. The fragmentation was performed on a Covaris device (KBioScience-LGC Genomics, Queens Road, Teddington, Middlesex, TW11 0LY, UK) through microTUBE and the size was read at 1.58 Kb on the Agilent 2100 BioAnalyzer on a DNA labchip High Sensitivity, as expected in the range of 1.5 Kb. The concentration of the shotgun library was measured on the fluorometer TBS and determined at 2.02E + 09 molecules/μL. The paired-end library was constructed from 5 μg of DNA. It was mechanically fragmented on Covaris device through miniTUBE-Red 3 Kb. The DNA fragmentation was visualized using the Agilent 2100 BioAnalyzer on a DNA labchip 7500 with an optimal size of 3 kb. The library was constructed according to the 454_Titanium paired end protocol and manufacturer (Roche). Circularization and nebulization were performed and generated a pattern with an optimal at 440 bp, respectively. After PCR amplification through 17 cycles followed by double size selection, the single stranded paired end library was quantified on the RNA pico 6000 labchip on the BioAnalyzer at 386 pg/μL. The library concentration equivalence was calculated at 1.61E + 09 molecules/μL. The library was stocked at -20 °C until used.

The XL+ shotgun library was clonal amplified with 0.5, 1 and 2 cpb in 2 emPCR reactions per conditions and the 3 kb paired end library was amplified with 0.5 and 2 cpb in 2 emPCR reactions per conditions and 4 reactions in 1cpb with the GS Titanium SV emPCR Kit (Lib-L) v2 (Roche). The yields of the emPCR were 5, 6.9 and 10 % respectively for the shotgun XL+, and 5.62, 10.27 and 14.92 % for the clonal amplification of the 3kb paired end libraries according to the quality expected by the range of 5 to 20 % from the Roche procedure. 790,000 beads of each library were loaded on a ¼ region from the GS Titanium PicoTiterPlate PTP Kit with the GS Titanium Sequencing Kit XLR70.

The runs were performed overnight and then analyzed on the cluster through the gsRunBrowser and gsAssembler_Roche. The global 566 858 passed filter sequences generated 201.6 Mb with a length average of 409 bp. These sequences were assembled on the gsAssembler from Roche with 90 % identity and 40 bp as overlap. It lead to 22 scaffolds, 417 large contigs (>1,500 bp) and 475 all contigs (>0.5 kb) and generated a genome size of 9.249 Mb which corresponds to a coverage of 22.4x equivalent genome.

### Genome annotation

Open Reading Frames were predicted using CLC Genomics Workbench software package 6.0.1 (CLC, Denmark). From the 417 contigs, any ORF spanning a sequencing gap region was eliminated. As for the Clusters of Orthologous Groups, rpsblast was done by blasting all predicted proteins against the National Center for Biotechnology Information (NCBI) COG database with an e-value of 10^-3^. The search for tRNA genes, ribosomal RNAs, proteins and genes predictions was completed by XEGEN. Phage detection was realized using the PHAST software [28], anti-smash 2 [29] for secondary metabolite detection, Resfinder tool [30] for antibiotic resistance genes, CRISPR Finder for clustered regularly interspaced short palindromic repeats [31] and GGDC web server [32] for in silico determining of DNA-DNA hybridization (DDH) values. Cell-division and cytoskeleton-related proteins were searched by running a blastp against a database described in [33], complemented by the FtsZl1 sequences [34]. We also targeted the peptidoglycan synthesis genes as described precedently [35]. A *G. massiliana* suspension, with an optical density of 1,1 at 260 nm when diluted 20 times, was required to conduct the pulsed-field gel electrophoresis and the southern blotting as previously described [36]. Migration parameters where fixed as following; initial time: 5 s, final time: 20 s, run time: 20 h, voltage: 5 V/cm, angle: 120°.

## Genome properties

**Fig. 6:**
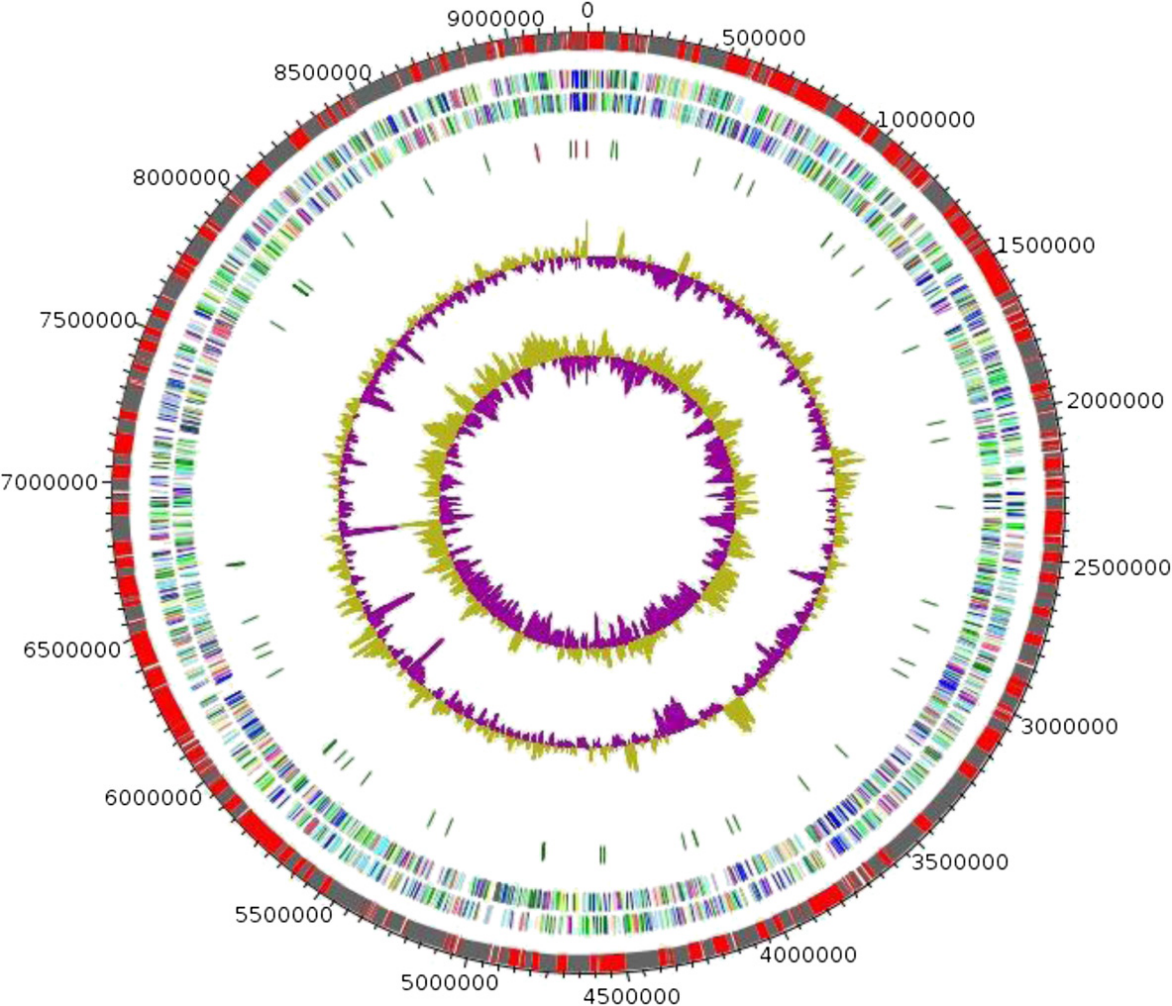
Graphical circular map of the chromosome. From outside to the center: Genes on the forward strand colored by COG categories (only genes assigned to COG), genes on the reverse strand colored by COG categories (only gene assigned to COG), RNA genes (tRNAs green, rRNAs red), GC content and GC skew

The genome is 9,249,437-bp long with 64.07 % GC content (Fig. 6). It is composed of 417 contigs (22 scaffolds). Of the 8,065 predicted genes, 7,985 were protein-coding genes, and 80 were RNAs (2 genes are 5S rRNA, 1 gene is 16S rRNA, 1 gene is 23S rRNA, 76 genes are tRNA genes). A total of 3,890 genes (48.72 %) were assigned a putative function. 1,097 genes were identified as ORFans (13.74 %). The remaining genes were annotated as hypothetical proteins (2,630 genes = > 32.94 %). The distribution of genes into COGs functional categories is presented in Table 3. The properties and the statistics of the genome are summarized in Tables 3 and 4.

**Table 3:** Genome statistics

Attribute	Value	% of total
Genome size (bp)	9,249,437	100
DNA coding (bp)	7,995,313	86.44
DNA G + C (bp)	5,925,688	64.07
DNA scaffolds	22	100
Total genes	8,065	100
Protein-coding genes	7,985	99.01
RNA genes	80	0.99
Pseudo genes	NA	-
Genes in internal clusters	3497	43.79
Genes with function prediction	3,237	40.53
Genes assigned to COGs	2,443	30.59
Genes with Pfam domains	6,088	76.24
Genes with signal peptides	1,979	24.78
Genes with transmembrane helices	1,515	18.97
CRISPR repeats	26	100

The total is based on either the size of the genome in base pairs or the total number of protein coding genes in the annotated genome

**Table 4:** Number of genes associated with general COG functional categories

Code	Value	%age	Description
J	168	2.10	Translation, ribosomal structure and biogenesis
A	1	0.01	RNA processing and modification
K	360	4.51	Transcription
L	404	5.06	Replication, recombination and repair
B	2	0.3	Chromatin structure and dynamics
D	28	0.35	Cell cycle control, mitosis and meiosis
Y	0	0	Nuclear structure
V	98	1.23	Defense mechanisms
T	419	5.25	Signal transduction mechanisms
M	260	3.26	Cell wall/membrane biogenesis
N	94	1.18	Cell motility
Z	0	0	Cytoskeleton
W	1	0.01	Extracellular structures
U	99	1.24	Intracellular trafficking and secretion
O	170	2.13	Posttranslational modification, protein turnover, chaperones
C	240	3.01	Energy production and conversion
G	240	3.01	Carbohydrate transport and metabolism
E	277	3.47	Amino acid transport and metabolism
F	63	0.79	Nucleotide transport and metabolism
H	130	1.63	Coenzyme transport and metabolism
I	125	1.57	Lipid transport and metabolism
P	188	2.35	Inorganic ion transport and metabolism
Q	143	1.79	Secondary metabolites biosynthesis, transport and catabolism
R	766	9.59	General function prediction only
S	368	4.61	Function unknown
-	4136	51.80	Not in COGs

The total is based on the total number of protein coding genes in the annotated geno

## Insights from the genome sequence

An incomplete 19.6-Kb phage sequence was detected, which lacks the attachment sites. A total of 21 questionable CRISPRs and 5 confirmed ones were found in the genome.At least 3 CAS proteins (CRISPR associated proteins) were also detected. No antibiotic resistance genes could be spotted using the resfinder tool, but the 15 clusters of secondary metabolites consisting of 5 terpene clusters, 5 bacteriocin clusters, 3 type four polyketide synthase(T4PKS) clusters, 1 T4PKS-T1PKS cluster, and 2 T3PKS clusters, put *G. massiliana* in the frontline of the *Planctomycetes* phylum, followed by *Schlesneria paludicola* with 13 clusters. As for the search of cell division- and cytoskeleton-related planctomycetal proteins, 10 were identified: FtsK, Noc, divK, divJ, FtsZl1, MraW, ClpX, CLpP, EnvA and FtsE. A plasmid replication protein with 44 % similarity to *Haliscomenobacter hydrossis* plasmid encoded RepA protein was predicted in the genome. Pulsed-field gel electrophoresis yielded slightly distinct bands so we decided to run a southern blot using genomic DNA and a DiG labeled DNA probe to try to confirm the presence of detectable plasmid, which was not the case. DDH values for 10 *Planctomycetes* genomes are presented in Additional file 1. DDH value between *G. massiliana* and *G. obscuriglobus* was 22.0 %.

While mining the genome for peptidoglycan synthesizing genes, only GT28 and GH73 genes were found. This is below the three-gene set previously shown to be associated with peptidoglycan synthesis [35], a minimal set of 3 genes is required for peptidoglycan metabolism. This observation agrees with the data available on *Planctomycetes* that lack peptidoglycan in their cell wall [37].

*G. massiliana* had a slightly larger genome than *G. obscuriglobus* (9.249 Mb vs 9.16 Mb), a lower G + C content (64 % vs 67.2 %), it codes for a higher number of genes (8,065 vs 7,645), had six cell-shape and division proteins in common with *G. obscuriglobus* and the other *Planctomycetes* previously studied [30] and four detected for the first time in this phylum. *G. massiliana* encodes 15 secondary metabolite gene clusters versus 12 in *G. obscuriglobus* and 26 CRISPRs versus 24 in *G. obscuriglobus*. It, also, showed a different antibiotic resistance profile [21].

## Conclusions

These results show that *G. massiliana* is a member of the genus *Gemmata*, exhibiting few features in common with the other characterized member of this genus, *G. obscuriglobus*. This unequivocally proves that *G. massiliana* is a new species of the genus *Gemmata*. Interestingly, *G. massiliana* exhibited two variants characterized by electron microscopy and FACS scan analysis a feature which has not been previously reported in other *Planctomycetes*. Nevertheless, we did observe this feature in another water-borne, not related β-*Proteobacteria*, *Minibacterium massiliensis* [38]. DDH *in silico* analysis revealed that differentisolates of a same *Planctomycetes* species exhibited a 66.7 % value, whereas isolates belonging to the same genus exhibited DDH values variation from 21.5 % DDH to 24.6 % DDH. Additionally, isolates affiliated to different genera exhibited DDH values variation from 17.5 % to 24 %, with the exception of *Rhodopirellula baltica**.* The value of *in silico* hybridization of *G. massiliana* with *G. obscuriglobus* was much lower than the 70 % DDH limit for delineating same species. These data confirm that *G. massiliana* should be considered as a new *Gemmata* species.

## Description of *Gemmata massiliana* sp. nov.

*Gemmata massiliana* (ma.si.lia.na L. fem. adj. of *Massilia*, taken from the old Greek and Roman name for Marseille where *Gemmata massiliana* was first isolated.) Pink colonies grown on IL agar varied in diameter between 0.1 mm and 1 mm. Optimal growth was observed at 30°C. Cells are gram negative cocci, motile, aerobic, with two cell populations of 1.1-μm and 2.1-μm diameter. Optimal growth occurs in absence of salt. The isolate tested negative for catalase and oxydase and positive for esculinase, alkaline phosphatase, naphtol-AS-BI-phosphohydrolase, valine, trypsin, acid phosphatase and leucine arylamidase. It was resistant to penicillin G, imipenem and amphotericin B. *G. massiliana* exhibited intermediate susceptibility to chloramphenicol, colistin, and is susceptible to tetracycline, ciprofloxacin, cotrimoxazole, gentamicin, minocycline, erythromycin and rifampicine.

The genome size is 9.249 Mb with a 64.07 % G + C content and 8065 predicted genes. Genome analysis identified an incomplete phage sequence, 26 CRISPRs and 3 CAS proteins, 15 clusters of secondary metabolites and 10 cell division- and cytoskeleton-related planctomycetal proteins: FtsK, Noc, divK, divJ, FtsZl1, MraW, ClpX, CLpP, EnvA and FtsE. Nonetheless no antibiotic resistance genes have been detected. The 16S rRNA gene and genome sequences have been deposited in GenBank under accession numbers JX088244 and CBXA010000001-CBXA010000171, respectively. The type strain (CSUR P189^T^, DSMZ 26013^T^) was isolated from a hospital water network.

## Additional files

Additional file 1:
**DDH values for 10** ***Planctomycetes*** **genomes.** Values are expressed in percentage. (XLS 26 kb)

## Abbreviations

ML: Marine-like
IL: *Isosphaera*-like
DDH: DNA-DNA hybridization
